# Nanotechnology Promotes Genetic and Functional Modifications of Therapeutic T Cells Against Cancer

**DOI:** 10.1002/advs.201903164

**Published:** 2020-02-20

**Authors:** Ahmed M. E. Abdalla, Lin Xiao, Yu Miao, Lixia Huang, Gendeal M. Fadlallah, Mario Gauthier, Chenxi Ouyang, Guang Yang

**Affiliations:** ^1^ Department of Biomedical Engineering College of Life Science and Technology Huazhong University of Science and Technology Wuhan 430074 China; ^2^ Department of Biochemistry College of Applied Science University of Bahri Khartoum 1660/11111 Sudan; ^3^ Department of Vascular Surgery General Hospital of Ningxia Medical University Yinchuan 750004 China; ^4^ Hubei Key Laboratory of Purification and Application of Plant Anti‐Cancer Active Ingredients School of Chemistry and Life Sciences Hubei University of Education Wuhan 430205 China; ^5^ Department of Chemistry and Biology Faculty of Education University of Gezira Wad‐Medani 2667 Sudan; ^6^ Department of Chemistry University of Waterloo Waterloo N2L 3G1 Canada; ^7^ Department of Vascular Surgery Fuwai Hospital National Center for Cardiovascular Disease Chinese Academy of Medical Sciences and Peking Union Medical College Beijing 100037 China

**Keywords:** cancer immunotherapy, chimeric antigen receptor T cell therapy, nanotechnology, T cell engineering

## Abstract

Growing experience with engineered chimeric antigen receptor (CAR)‐T cells has revealed some of the challenges associated with developing patient‐specific therapy. The promising clinical results obtained with CAR‐T therapy nevertheless demonstrate the urgency of advancements to promote and expand its uses. There is indeed a need to devise novel methods to generate potent CARs, and to confer them and track their anti‐tumor efficacy in CAR‐T therapy. A potentially effective approach to improve the efficacy of CAR‐T cell therapy would be to exploit the benefits of nanotechnology. This report highlights the current limitations of CAR‐T immunotherapy and pinpoints potential opportunities and tremendous advantages of using nanotechnology to 1) introduce CAR transgene cassettes into primary T cells, 2) stimulate T cell expansion and persistence, 3) improve T cell trafficking, 4) stimulate the intrinsic T cell activity, 5) reprogram the immunosuppressive cellular and vascular microenvironments, and 6) monitor the therapeutic efficacy of CAR‐T cell therapy. Therefore, genetic and functional modifications promoted by nanotechnology enable the generation of robust CAR‐T cell therapy and offer precision treatments against cancer.

## Graphical abstract

Herein, the current limitations of chimeric antigen receptor (CAR)‐T immunotherapy and potential opportunities and tremendous advantages of using nanotechnology to optimize CAR‐T cell therapy against cancer are highlighted. The genetic and functional modifications promoted by nanotechnology‐based strategies enable the generation of robust CAR‐T cell therapies and offer precision treatments against cancer.

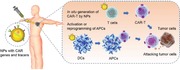

## 1 Introduction

Decades of research and clinical investigations have led to significant advances in engineered chimeric antigen receptor (CAR) T cell immunotherapy.^[^ 1, 2, 3 ^]^ Besides positive clinical results obtained in the treatment of B‐cell malignancy, several spectacular endeavors are in progress to expand the use of CARs in the treatment of solid tumors and other clinical conditions such as autoimmune and infectious diseases.^[^ 4, 5, 6 ^]^ Despite these advances, summarized in **Table** **1**, CAR‐T cell therapy still faces several major issues to be addressed with respect to the preparation of CAR‐T cells, safety concerns, and therapeutic efficacy, especially against solid tumors.

**Table 1:** Summary of most significant CAR‐T cell therapy advances. Adapted from the Annual Report of the American Society of Clinical Oncology (ASCO), 2018^[^ 1 ^]^

Disease	CAR‐T cell therapy	Response ratio (period)	Remission ratio	Ref.
ALL	Kymriah (CTL019)	90% (5 years after diagnosis)	82%	^[^ 7 ^]^
DLBCL	Kymriah (CD19)	80% (6 month after treatment)	43%	^[^ 8 ^]^
Refractory NHL	Yescarta (ZUMA‐1)	40% (9 month after treatment)	54%	^[^ 9 ^]^
Multiple myeloma	BCMA CAR‐T	31% (>6 months after treatment)	94%	^[^ 10 ^]^

ALL, acute lymphoblastic leukemia; DLBCL, diffuse large B‐cell lymphoma; NHL, non‐Hodgkin lymphoma; BCMA, B‐cell maturation antigen.

**Figure 1:**
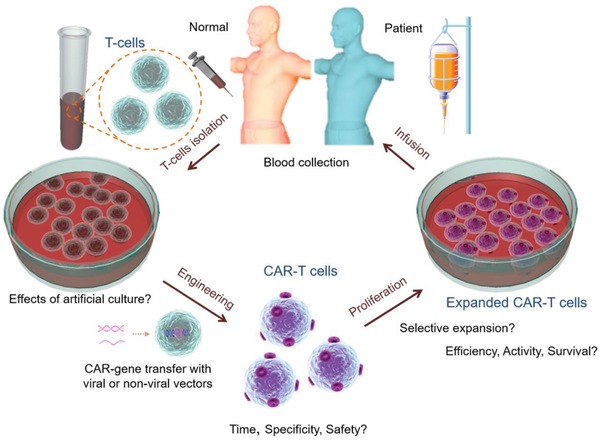
Steps in the production of CAR‐T cells. This usually starts with drawing blood from the patient, followed by T cell isolation. The T cells are then cultivated for some time, genetically engineered to produce specific tumor cell‐surface receptors or antigens containing the CAR sequence. After processing, the genetically modified T cells are selectively multiplied and infused back into the patient.

CAR‐T cells for clinical uses are generated through a complex ex vivo approach,^[^ 11, 12 ^]^ as shown in **Figure** **1**. The first step of this approach is drawing blood from the patients, followed by the isolation of a sufficient number of T cells. The cells are then processed by genetic engineering, either with viral or non‐viral vectors, which represents the most critical manufacturing step, to produce specific tumor cell‐surface receptors or antigens containing CAR transgene cassettes.^[^ 13 ^]^ These transfection procedures may be associated with serious side effects or complications.^[^ 14 ^]^ Other factors such as the selection of suitable T cell phenotypes, the cultivation of T cells for several weeks to generate a sufficient number of CAR‐T cells, and substrate rigidity in artificial cultures may also affect the characteristics of CAR‐T cell products and induce T cell exhaustion, apoptosis, and reduction.^[^ 15 ^]^

**Figure 2:**
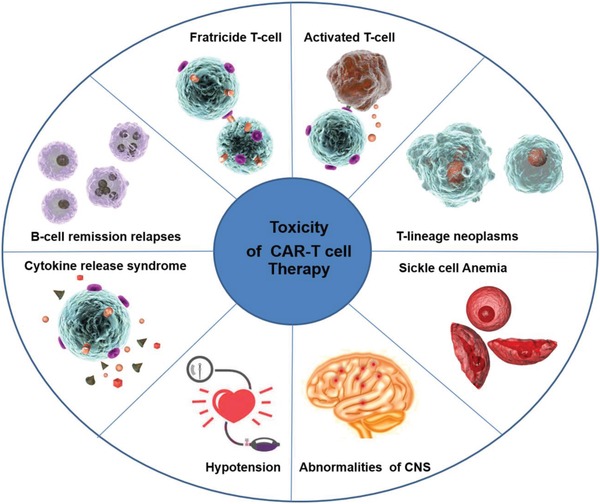
Depiction of reported toxicities in CAR‐T cell therapy. “On target, off tumor” toxicity potentially leading to profound fratricide T cells and hyperimmune activation, for example, the cytokine release syndrome (CRS), while cross‐reactivity leads to B‐cell remission relapses and T‐lineage neoplasms. Other serious side effects include abnormalities in the central nervous system (CNS), arterial hypotension, sickle cell anemia, and sometimes organ damage.

Immune system mediated adverse effects of CAR‐T cell therapy are more serious than for conventional pharmaceutical treatments^[^ 16, 17 ^]^ and can lead to expected or unexpected toxicities, such as cross‐reactivity (killing of normal cells),^[^ 18 ^]^ potentially leading to “on target, off tumor,” profound immunodeficiency, or to the fratricide of CAR‐T cells (**Figure** **2**).^[^ 19, 20 ^]^ For example, patients who received CAR‐T cell therapy in recent clinical trials displayed problems associated with hyperimmune activation (e.g., cytokine release syndrome, CRS), abnormalities of the central nervous system (CNS), arterial hypotension, and organ damage.^[^ 3, 18, 21, 22, 23 ^]^ Further investigations are clearly required before CAR‐T cell immunotherapy can be exploited as a broad therapeutic strategy.

Critical issues limiting the anti‐tumor efficacy of CAR‐T cell therapy are tumor resistance,^[^ 24 ^]^ tumor antigen escape relapse,^[^ 25 ^]^ and the suppressive nature of the tumor microenvironment (TME),^[^ 26, 27 ^]^ characterized by large gradients in cell proliferation rates, a high interstitial fluid pressure (IFP), and regions of hypoxia and acidosis.^[^ 28 ^]^ In addition to their roles in the suppression of immune response and the inhibition of cytotoxic T cell proliferation,^[^ 29 ^]^ these critical features of the TME allow tumors to actively escape T‐cell‐mediated tumor‐specific immunity, by activating negative regulatory pathways (checkpoints) such as programmed cell death protein‐1 (PD‐1).^[^ 30 ^]^ Abnormal tumor vessels (leaky, tortuous, and dilated blood vessels) may also create a physiological barrier to CAR‐T cell trafficking and infiltration,^[^ 31 ^]^ and facilitate immune evasion.^[^ 32 ^]^

Interestingly, the growing importance of CAR‐T cell therapy coincides with the maturation of nanotechnology and synthetic materials, and thus potent tools and approaches, discussed herein, are being utilized to produce robust effector CAR‐T cells by addressing the engineering requirements of adoptive T cells. In particular, nanoparticles (NPs), encompassing a broad assortment of biomaterials in the 10–100 nm range such as lipid‐based particles, polymeric NPs, and metallic and other inorganic NPs, present unique properties such as large surface area, shape, ultra‐small size, and ability to manipulate the surface with biological entities (e.g., proteins and genes). In addition to their ability to target immune cells and stimulate the innate immunity via toll‐like receptor (TLR) pathway, they can potentially serve to improve the outcome of genetically engineered T cell therapy against cancer.^[^ 33, 34 ^]^ Exposing therapeutic cells to NP reagents may help to mediate in vivo delivery of gene cargo into T cells without compromising their proliferation, improve gene silencing, enhance the activity, and improve the stability and therapeutic efficiency of T cells.^[^ 35 ^]^ Considering the intrinsic properties of NPs, these could serve to improve the delivery of immune modulators,^[^ 36 ^]^ prevent tumor relapse,^[^ 37 ^]^ and monitor the therapeutic response to cancer therapy.^[^ 38 ^]^

This report considers the benefits of NPs in CAR‐T cell therapy by i) promoting CARs generation, ii) enhancing the proliferation and survival of CAR‐T cells, iii) stimulating the intrinsic activity of CAR‐T cells, iv) improving CAR‐T cell trafficking, v) overcoming immunosuppressive tumor‐associated cells and vessels, and vi) monitoring the therapeutic fate of CAR‐T cells. Based on a thorough literature review and our expertise in this field, we realized that it is timely to reflect on the necessity and importance of nanotechnology applied to promoting cytotoxic T cell therapy, even though this approach has just emerged in recent years. The ideas discussed in this report, beyond reporting on the great progress in therapeutic T cell against cancer brought about by nanotechnology, should stimulate new developments in that area.

## 2 Nanoparticles Potentiate the Generation of CAR Transgene Cassettes in T Cells

**Figure 3:**
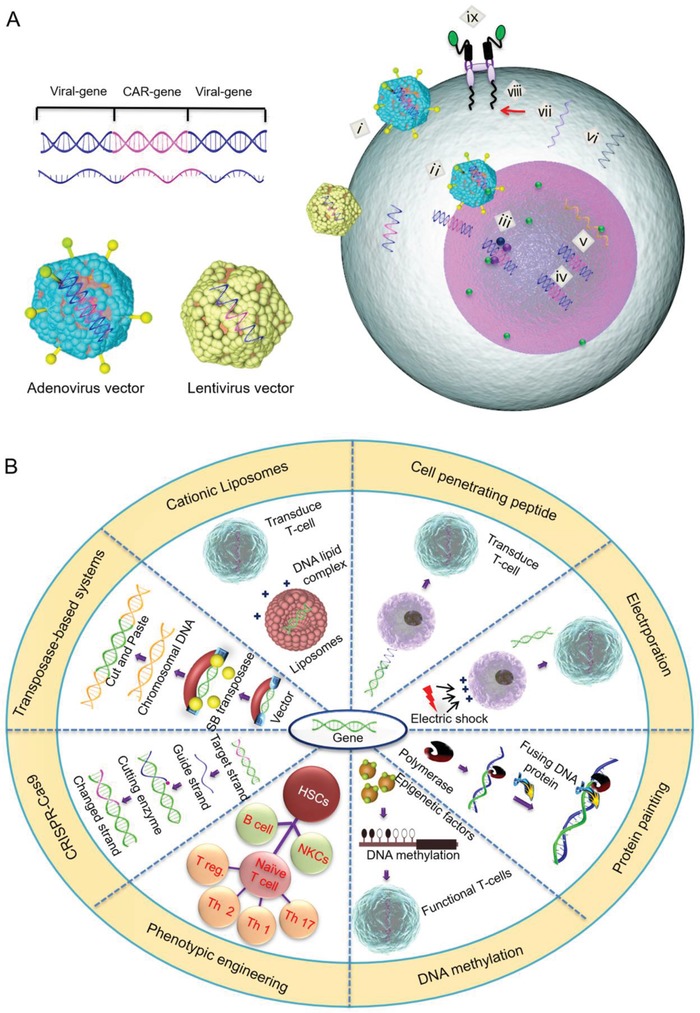
A) Steps in viral and nonviral‐mediated CAR transfer methods. After the integration of CAR encoding genes in virus vectors, the virus binds to the cell membrane (i) and releases the encoding gene into the nucleus (ii) by endocytosis so that it can integrate (iii), replicate (iv), and translate into mRNA (v), resulting in the internalization and expression of the CAR gene (vi–ix). B) Representative nonviral‐mediated CAR transfer strategies and associated mechanisms.

Genetically engineered T cell infusion in cancer patients was first investigated in 1990.^[^ 39 ^]^ Since then, a wide range of tools have been used to modify lymphocytes and lymphoid progenitors, so as to make them suitable for immunotherapy. The transfer of CARs into both autologous and allogeneic T cells has been investigated through multiple strategies, including viral (**Figure** **3**A) and non‐viral (Figure 3B) CAR transfer methods. Most of these genetic strategies are aimed to prevent T cell malfunction, and to increase the recognition of tumor antigens as well as antitumor immunity.^[^ 40 ^]^ Unfortunately, the generation of CARs through viral or non‐viral gene delivery systems suffers from several complications. This section will discuss how NPs can serve to promote CAR generation, to overcome the limitations, or improve the transfection efficiency of viral and non‐viral vectors.

### 2.1 Replacing Viral‐Mediated CAR Transfer Methods

Gene therapy tools such as viral vector based methods are effective and reliable techniques leading to prolonged expression of the desired transgene by T cells.^[^ 40 ^]^ As compared to early treatments using hematopoietic stem cells (HSCs), the viral‐based transduction of T cells was shown to have low adverse outcomes^[^ 41 ^]^ and low intrinsic immunogenicity.^[^ 42 ^]^ Lentivectors, γ‐retroviral, and other non‐integrating vectors^[^ 43 ^]^ were successfully used to transduce CARs into the T cells of patients.^[^ 44, 45, 46 ^]^ Unfortunately, the clinical uses of viral vectors are restricted by their high cost, the long time required for their implementation, and other safety concerns such as mutagenicity, the generation of infectious viruses, variegated transgene expression, clonal expansion, and transcriptional silencing (**Table** **2**).^[^ 47, 48, 49 ^]^ Furthermore, the expression of cancer receptor genes by T cells also requires complex systems to sustain and integrate the newly transferred genes into the DNA of the patient.^[^ 50, 51 ^]^ Briefly, the consequences of viral transfection can range from ineffectiveness in creating sustained transgene expression, to the generation of acute systemic toxicity, transformed cell growth, and even oncogenesis. As a result, there is a need for alternate transfection methods that are less immunogenic and more efficient.

**Table 2:** Generation of CAR‐T cells by viral vectors followed by fatal or serious adverse effects

Viral vector	Center	Disease	No. of patients/outcome	Adverse effects	Ref.
γ‐Retroviral	NCI	B lymphomas	8/6 remission	Tumor lysis	^[^ 52 ^]^
		Post‐stem cell transplantation	10/3 regression	B cell aplasia and hypotension	^[^ 53 ^]^
	Baylor	NHL	6/2 responses	Cytokine release syndrome	^[^ 54 ^]^
		Allo‐stem cell transplantation	8/2 responses	Cytokine release syndrome	^[^ 55 ^]^
	MSKCC	CLL	8/1 response	Death and B cell aplasia	^[^ 56 ^]^
		ALL	5/5 minimal residual disease	Cytokine release syndrome	^[^ 17, 57 ^]^
Lentiviral	UPENN	CLL	3/2 complete response and 1 partial response	Tumor lysis syndrome	^[^ 44, 58 ^]^

NCI, National Cancer Institute; NHL, non‐Hodgkin lymphoma; MSKCC, Memorial Sloan Kettering Cancer Center; CLL, chronic lymphocytic leukemia; ALL, acute lymphocytic leukemia; UPENN, University of Pennsylvania.

It has been confirmed that NPs can achieve the targeted delivery of specific genes to human T cells to enhance immune activation.^[^ 59 ^]^ Moreover, as compared with viral transfection, the transfection efficacy of genes can be improved and their off‐target drawbacks can be alleviated by taking advantage of NPs.^[^ 35, 60 ^]^ These facts demonstrate the possibility of applying NPs as gene carriers to generate CARs in place of viral vectors. Recently, Smith and co‐workers successfully replaced lentivirals with polymeric NPs to generate CAR‐T cells in vivo, without removing them from the patient.^[^ 35 ^]^ The authors used several tricks to introduce CD19 CAR constructs into the nucleus of T cells selectively: First, the surface of polymeric NPs was functionalized with anti‐CD3e F(ab′)2 fragments to target T cells. Second, the cargo was delivered to the nucleus of the T cells in the presence of a nuclear localization sequence (NLS) and a microtubule‐associated sequence (MTAS). Finally, the CD19 CAR construct was flanked with piggyBac transposase elements and introduced into the DNA of the T cells through a cut‐and‐paste mechanism.^[^ 35 ^]^ Interestingly, the therapeutic efficacy of their nanoparticle system was similar to CAR‐T cells generated ex vivo in an animal model with CD19^+^ leukemia. Furthermore, they successfully established the advantages of NPs over viral vectors in delivering DNA cargo into T cells, by efficiently enabling CAR expression and promoting the in vivo expansion of CD19 CAR‐T cells. This in vivo expansion is technically obviously simpler than ex vivo expansion, and through this approach, Smith et al. evaded the complications of lengthy culturing, which can exhaust T cells and affect their efficacy over time.^[^ 35 ^]^

### 2.2 Optimization of Nonviral‐Mediated CAR Transfer Strategies

Besides generating CAR‐T cells in vivo by replacing ex vivo viral transfection, NPs have also shown great potential in optimizing nonviral‐mediated CAR transfer strategies, including chemical transfection techniques, electroporation, transposase‐based approaches, genome editing, and phenotypic engineering, as summarized in **Table** **3**.

**Table 3:** Representative NP‐based strategies to optimize CAR‐T cell therapy

Non‐viral approaches	Example of NP (or system)	Advantages	Remarks	Ref.
Cationic polymers	PHEMA‐*g*‐PDMAEMA NPsa)	Successful transfection of T cells with messenger RNA and plasmid DNA with low toxicity (>90% viability)	Optimized the primary T cell transfection conditions	^[^ 34 ^]^
Cationic liposomes	Lipid‐based NPs	Mediates in vivo nucleic acid delivery to T cells	T cell proliferation and cytolytic function not compromised	^[^ 61, 62 ^]^
Electroporation‐based method	Encapsulation of synthetic mRNA in polymeric PGA NPs	Specific cell subtype targeting, stimulation of receptor‐mediated endocytosis, improved therapeutic potential of programmed T cells	Successful removal of the TRAC region, an important challenge to optimize CAR‐T cell therapy	^[^ 63 ^]^
Transposon‐based integration	Polymeric NPs (anti‐CD3‐coupled PEI)	Efficient delivery of DNA cargo into T cells, CAR expression enabled, and in vivo expansion of CAR‐T cells promoted	Technically simpler and generation of potent CAR‐T cells inside the body	^[^ 35 ^]^
CRISPR‐CAS9 editing	CRISPR/Cas9‐RNP co‐engineered with nanoparticles	Increased delivery efficiency (up to ≈90%) and great promise for gene repair	Excellent non‐viral editing system reducing off‐target mutations	^[^ 64, 65 ^]^
Phenotypic changes	Encoding mRNA for transcriptional factor Foxo13A in NP system	Provided effective immune response	Improved activity of CAR‐T cells in B‐cell lymphoma animal models	^[^ 66 ^]^
Epigenetic‐based method	Nanocomplex of miR‐155 mimics and PEI NPs	Reprogramming of tolerogenic DCs into immunostimulatory cells	Potent stimulation of T cell activation	^[^ 67 ^]^

a) Poly(hydroxyethyl methacrylate)‐graft‐poly(2‐(dimethylamino)ethyl methacrylate).

Chemical transfection techniques, for example, using cationic polymers, cationic liposomes, and peptides, are typical transfection methods for common cell lines, and can achieve efficient gene transfer with relatively low cytotoxicity.^[^ 68 ^]^ Unfortunately, the transfection efficiency of these systems varies with the cell type, the cell membrane condition, the pH, and the nucleic acid to chemical ratio.^[^ 69 ^]^ Nanotechnology‐based approaches have also been used to transfect primary immune cells effectively. For instance, the transfection of primary and cultured human T cells by a range of cationic polymers, including comb‐ and sunflower‐shaped poly(hydroxyethyl methacrylate)‐grafted poly(2‐(dimethylamino)ethyl methacrylate) NPs was evaluated.^[^ 34 ^]^ The results obtained demonstrated that after optimizing the primary transfection conditions for the T cells (culture medium, cell density, activation time, cytokine treatment, and DNA dose), CD4^+^ and CD8^+^ primary human T cells could be successfully transfected with messenger RNA (25% efficiency) and plasmid DNA (18% efficiency) with minimal concomitant toxicity (>90% viability). Similarly, high (50%) transfection efficiency and viability were achieved in Jurkat human T cell lines, again confirming the potential of these NPs.^[^ 34 ^]^ Furthermore, it was shown that the attachment of siRNA‐loaded liposomes to the surface of cytotoxic CD8^+^ T cells can mediate the in vivo delivery of siRNA to T cells without compromising their proliferation and cytolytic function.^[^ 61, 62 ^]^

The RNA‐based electroporation of lymphocytes is another attractive approach offering the potential to promote CAR design and potency.^[^ 70 ^]^ An in vitro investigation thus indicated that transcribed mRNA induced transient protein expression and redirected the transduced T cells (with up to 100% efficiency) for the RNA encoding of CARs.^[^ 71 ^]^ Based on this concept, Moffett et al. effectively removed the T cell receptor alpha constant region (TRAC) using megaTAL nuclease and abolished the production of non‐cancer‐specific T cell receptors (TCRs), which is an important challenge in improving CAR‐T cell therapy.^[^ 63 ^]^ To achieve their goal, they delivered mRNA genes to T cells by targeting CD3‐ and CD8‐cells. Their results confirmed that the addition of bioengineered NPs can serve to target specific cell subtypes, stimulate receptor‐mediated endocytosis (namely, the “enhancement of RNA entry by a physiological process without compromising cell viability”), transiently program the expression of genes, and improve the therapeutic potential of both programmed T cells and stem cells. Furthermore, in a photoporation‐based study, the addition of gold nanoparticles (AuNPs) to CD8^+^ cytotoxic T cell (CTL) was used to achieve transient permeabilization. This approach was characterized by a lower cytotoxicity as compared to nucleofection with similar siRNA‐mediated gene knockdown.^[^ 66 ^]^ Other electroporation approaches, such as cell‐penetrating peptides (CPPs), were used to enhance the cellular uptake of different therapeutic cargos and transport them into cells.^[^ 72 ^]^ It was further reported that the incorporation of magnetic Fe_3_O_4_ NPs into CCP‐oligonucleotide complexes can promote cell transfection and improve gene silencing, splice correction, and plasmid transfection.^[^ 73 ^]^

Various transposon‐based systems with distinctive DNA elements that have capacity to travel in different chromosomal locations such as Sleeping Beauty (SB), Tol2, and piggyBac (PB) were developed to engineer T cells.^[^ 74, 75, 76 ^]^ In a recent clinical trial, the SB transposon system was found to be safe to generate CAR‐T cells.^[^ 77 ^]^ Despite these advances, several challenges still need to be addressed such as the optimization of the transposon‐to‐transposase ratio, which can otherwise lead to a phenomenon termed “overproduction inhibition.”^[^ 78 ^]^ Another concern is related to the uptake of transposon/transposase constructs into lymphoid T cells at a level sufficient to achieve high and stable therapeutic gene expression.^[^ 79 ^]^ However, even if sustained transposase expression is achieved, a high genotoxic risk may exist due to random transposition of the inserted transposons.^[^ 80 ^]^ Consequently, the transposon systems are not yet sufficiently potent for gene therapy. A combination of nanotechnology with the piggyBac transposon delivery system has been explored in humans by Smith et al.^[^ 35 ^]^ Besides promising results, this combination is preferred because human cells can re‐locate piggyBac transposons carrying the inserted genes, owing to the presence of endogenous transposase activity on these cells.^[^ 35 ^]^

Genome editing tools based upon programmable nucleases such as the CRISPR (clustered regularly interspaced short palindromic repeats), TALEN (transcription activator like effector‐based nucleases), and ZFN (zinc finger nucleases) have the ability to induce considerable changes in the genome of eukaryotic cells.^[^ 81 ^]^ Relative to non‐gene‐edited T cells, gene‐edited T cells were confirmed to have robust antitumor activity and an improved biosafety profile in both clinical trials^[^ 82 ^]^ and different animal models.^[^ 83 ^]^ Furthermore, gene‐edited CD19 CAR‐T cell therapy was shown to lead to complete remission for 30% of patients with B‐cell malignancies, without causing graft‐versus‐host disease or infusion‐related toxicity.^[^ 84 ^]^ While the CRISPR‐Cas9 technology offers tremendous promise, it is still in early stages of development and a number of issues need to be optimized such as technical complications, safety, editing ability, and the efficient release of CRISPR‐Cas9 components to their targets in vivo.^[^ 85 ^]^ The integration of nuclease DNA or RNA leads to increased genome editing ability, through the constitutive expression of nucleases, but also increases the chances of off‐target mutations.^[^ 86 ^]^ It was shown that the direct cytoplasmic/nuclear delivery of CRISPR/Cas9‐ribonucleoprotein (Cas9‐RNP) co‐engineered with carrier nanoparticles led to enhanced delivery efficiency (≈90%) and reduced off‐target mutations in gene delivery strategies.^[^ 65 ^]^ Furthermore, gold NP‐based CRISPR‐Cas 9 was shown to be an excellent non‐viral editing system with great promise for gene repair in future clinical applications.^[^ 64 ^]^

Phenotypic engineering is an additional strategy that can be accomplished through the transient expression of different genes designed to achieve molecular programming, such as the “hit‐and‐run” gene‐targeting technique.^[^ 87 ^]^ Interestingly, phenotypic changes were efficientl*y* achieved by Wayteck et al. in a novel approach by which central memory T cells were enriched by inserting encoding mRNA for transcriptional factor Foxo13A into an NP system to target CD3.^[^ 66 ^]^ The treatment of T cells by this method provided effective immune response and improved the activity of CAR‐T cells in B‐cell lymphoma animal models.

## 3 Nanoparticle‐Based Gene Delivery Induces the Efficiency of CAR‐T Cells

The expansion of immune cells is an essential process to maintain the number of periphery cells and accurately represent both naïve and memory cells for sustained proliferation. Moreover, immune cell expansion upon antigen contact is a key step in the modulation of immune response to cytokines and infections.^[^ 88 ^]^ Clinical evidence from CAR‐T cell therapy has shown the absolute clinical significance, in both hematological and solid cancer patients in particular, of T cell expansion and long‐term persistence.^[^ 89 ^]^ In addition to cell expansion and persistence inside tumors, the trafficking and activity of CAR‐T cells in tumor sites are significant issues for solid tumors. It seems likely that advances in nanotechnology could be harnessed in novel ways so as to enhance CAR‐T cell expansion, persistence, trafficking, and activity. These facts are discussed in the following sections.

### 3.1 Promotion of CAR‐T Cell Expansion and Persistence

In the case of hematological cancer, when CD19 CAR‐T cells are infused, they initially encounter CD19 targets and start to be activated and expand.^[^ 3 ^]^ However, the question remains as to what happens in the case of solid tumors. Are T cells sufficiently expanded to eliminate the tumor? Do CAR‐T cells persist long enough to remove the tumor? Improvement in CAR‐T cell proliferation is thus a critical challenge. Furthermore, the expansion of effector immune cells without apoptosis is another task for adaptive T‐lymphocytes and must be considered seriously to avoid offensive immune cell activation, which may cause chronic inflammation, allergic or autoimmune disorders, and ultimately may influence the therapeutic intervention either positively or negatively.^[^ 90 ^]^

Nanotechnology could be exploited to stimulate CAR‐T cell expansion and persistence without detectable toxicity. It was indeed shown that CAR‐T cell expansion could be potently enhanced in vitro and in vivo using advanced nanosystems.^[^ 35 ^]^ For example, Darrell et al. designed novel cell surface conjugated nanogels with interleukin‐15 super‐agonist to “backpack” a considerable quantity of protein drugs into T cells.^[^ 91 ^]^ The NG system selectively released its protein cargo, depending on T cell receptor activation, achieving controlled drug release to antigen encounter sites such as the TME. Besides its selectivity, the system specifically promoted T cell expansion 16‐fold at tumor sites and permitted the administration of cytokine at 8‐fold higher doses without toxicity.

Another promising way to enhance T cell expansion is using artificial substrates to attach T cell stimuli. Using this concept, T cell expansion was stimulated with carbon nanotube–polymer composites as synthetic antigen‐presenting cells (APC).^[^ 92 ^]^ The investigators used bundled carbon nanotubes to attach the antigens, and then combined this complex with magnetite–polymeric NPs in the presence of a specific T cell growth factor such as interleukin‐2 (IL‐2), required for immune response and T cell proliferation. The expanded T cells obtained with this system were compared with clinical standards, confirming that this composite had the ability to reproduce potent cytotoxic T cells for cancer therapy.

### 3.2 Modulation of the Trafficking and Potency of CAR‐T Cells

A number of tumors are indeed characterized by the presence of fibrotic cells which may physically hinder T cell penetration. Other tumors may adopt features such as low T cell infiltration, or reprogram themselves to actively escape T‐cell‐mediated tumor‐specific immunity by triggering the immune checkpoint molecules.^[^ 30 ^]^ The seminal discovery of checkpoints, namely PD‐1 and cytotoxic T‐lymphocyte‐associated antigen‐4 (CTLA‐4), by Honjo and Allison (Nobel Prize winners, 2018), respectively, established a novel principle for understanding the suppressive nature of tumor cells.^[^ 93, 94 ^]^ Indeed, the activation of checkpoint inhibitors effectively suppresses the CAR‐T cell trafficking and activity, and even the efficacy of CAR‐T cell therapy in cancer patients who fail to respond to CD19 CAR‐T cells alone.^[^ 95, 96, 97 ^]^

**Figure 4:**
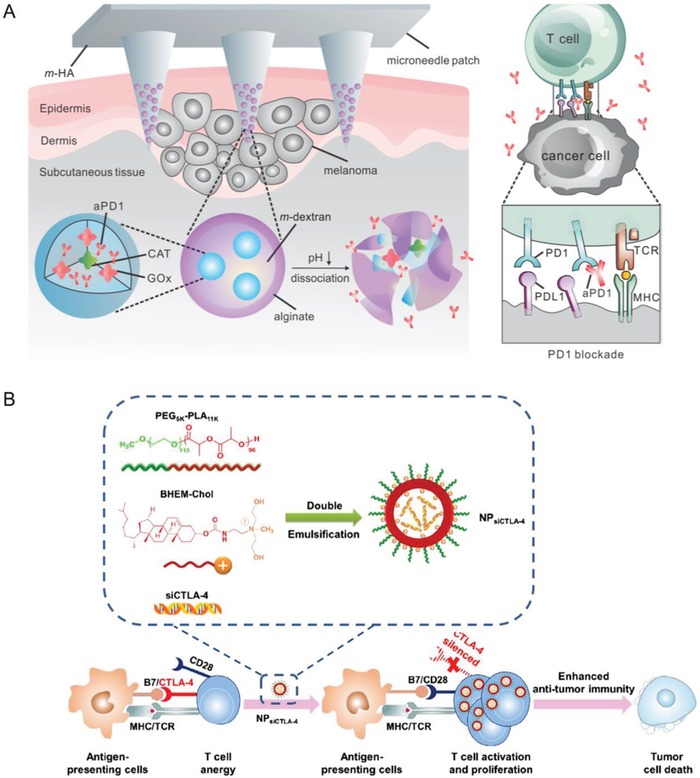
Nanoparticle‐mediated immune checkpoint modulation to enhance anti‐tumor functions of T cells. A) Schematic of the microneedle patch‐assisted delivery of aPD1 for the skin cancer treatment. The aPD1 and GOx/CAT enzymatic system were loaded inside the NPs and delivered by a microneedle patch. The enzyme‐mediated conversion of blood glucose to gluconic acid promotes the sustained dissociation of NPs, subsequently leading to the release of aPD1. The immune system was activated to destroy skin cancer cells through the blockade of PD‐1 by aPD1. Reproduced with permission.^[^ 100 ^]^ Copyright 2016, American Chemical Society. B) Enhanced T‐cell‐mediated immune responses and anti‐tumor efficacy were achieved by blocking CTLA‐4 using siCTLA‐4‐encapsulated nanoparticles (NP siCTLA‐4). NP siCTLA‐4 was prepared with poly(ethylene glycol)‐*block*‐poly(d,l‐lactide) and a cationic lipid BHEM‐Chol by double emulsification. Reproduced with permission.^[^ 102 ^]^ Copyright 2016, Elsevier.

To solve such challenges, different NP‐based approaches have been developed to release immunostimulatory cytokines, or produce armored CAR‐T cells, a neutralized scFvs directed against checkpoint inhibitors.^[^ 26, 98, 99 ^]^ For instance, Gu et al. designed self‐degradable microneedle patch‐coupled immune checkpoint inhibitor (anti‐PD1 antibody, aPD1) delivery approaches to treat skin cancer^[^ 100 ^]^ (**Figure** **4**A). The microneedle patch was conjugated with aPD1, dextran NPs as pH‐detector, and glucose oxidase (GOx) for sustained drug release. Alginate was also integrated with the NPs, to coat their surface with negative charges. The NPs (250 nm) were successfully introduced in microneedles, and the aPD1 was safely released using a glucose‐specific enzyme. This system effectively stimulated trafficking of both CD4^+^ and CD8^+^ T cells and inhibited tumor growth.

In another study, Gu et al. improved the targeting of anti‐programmed cell death protein ligand‐1 (PD‐L1) antibody through conjugation with platelet‐derived microparticles (PMPs).^[^ 101 ^]^ This approach likewise enhanced CD4^+^ and CD8^+^ T cell trafficking, reduced cancer growth and metastasis, and prolonged the survival of tumor‐bearing mice (B16‐F10 and 4T1). Moreover, it was shown that the encapsulation of PD‐L1 siRNA in folic acid modified poly(ethylene imine) (PEI) downregulated PD‐L1 in SKOV‐3‐Luc tumor cells, resulting in enhanced T cell sensitization of tumors in vitro and the mediation of immune responses in vivo.

Along the same line, Wang et al. synthesized poly(ethylene glycol)‐*block*‐poly(l‐lactide) (PEG‐*b*‐PLA) NPs and then encapsulated CTLA‐4 siRNA (siCTLA‐4), for immune checkpoint modulation, with *N*‐bis(2‐hydroxyethyl)‐*N*‐methyl‐*N*‐(2‐cholesteryloxycarbonyl aminoethyl) ammonium bromide into PEG‐*b*‐PLA NPs by a water‐in‐oil‐in‐water emulsion technique^[^ 102 ^]^ (Figure 4B). The integrated NPs (NPsiCTLA‐4) were able to introduce the siRNA into T cells, knocking down the CTLA‐4 mRNA and protein levels in activated T cells in vitro. Furthermore, the systemic delivery of NPsiCTLA‐4 increased the trafficking rate of both CD4^+^ and CD8^+^ T‐lymphocytes, with a decreased extension ratio for CD4^+^ FOXP3^+^ regulatory T cells, resulting in tumor growth inhibition.

The targeted delivery of immune stimulatory materials to cytotoxic CD8^+^ T cells by smart NP systems appears to be another promising strategy.^[^ 103 ^]^ Goldberg et al. thus synthesized anti‐PD1 fragment‐coupled NPs prepared from poly(lactic acid‐*co*‐glycolic acid)‐*block*‐poly(ethylene glycol) (PLGA‐*b*‐PEG), an FDA‐approved copolymer. To target endogenous CD8^+^ T cells in the blood and tumors, the surface of these NPs was functionalized with CD8‐fragmented antibody (Fab). The NPs were then conjugated with PD‐1 antibody (PD‐1‐PLGA‐*b*‐PEG) to selectively target PD‐1^+^ T cells. Furthermore, dithiothreitol (DTT) was used to reduce specific site‐cleaved aPD1 fragments after conjugation with the surface of PLGA‐*b*‐PEG NPs. As compared with the systemic administration of free drug, these NPs efficiently stimulated T cell functions, with selective and robust targeting in vitro and in vivo. The PLGA‐*b*‐PEG NP system was co‐encapsulated with a transforming growth factor‐β (TGF‐β) inhibitor, resiquimod (R848), to stimulate the proliferation of tumor‐infiltrating CD8^+^ T cells, or with SD‐208, to sustain the release of the drug in systemic circulation. The encapsulation of SD‐208 in the PLGA‐*b*‐PEG constructs led to significant inhibition of non‐specific toxicity linked with autoimmune response. In addition, these NPs dramatically reduced tumor growth, prolonged survival in cancer animal models, and mitigated other drawbacks of cancer immunotherapy. Kuai et al. prepared nanodiscs (high‐density lipoproteins conjugated with antigen peptides and adjuvants) to target patient‐selective neoantigens and enhance potent CD8^+^ cytotoxic T cell response in melanoma.^[^ 104 ^]^ Interestingly, their system produced much (47‐fold) more CTL‐selective neoantigen than soluble vaccines, as compared with a 31‐fold improvement for clinically approved Montanide with CpG as adjuvant. A combination therapy of nanodiscs and anti‐PD‐1 aggressively eliminated the tumors. Owing to the smaller size (≈20 nm) of the nanodiscs, the stability of antigen peptides in vivo, and efficient antigen expression in APCs, these results suggest that potent anti‐tumor immune response can be achieved through this novel approach to personalized nanomedicine.

Although the combination of CAR‐T cell therapy with immune checkpoint inhibitors has yielded promising results in cancer treatment,^[^ 105 ^]^ immunosuppressive tumor microenvironments and microvasculature may counter the trafficking and activity of T cells and critically inhibit their anti‐tumor effects in certain settings.^[^ 27, 29 ^]^

## 4 Nanoparticles as a Potent Platform for Reprogramming Tumor‐Associated Cells and Vessels

Given the essential role of cancer‐associated cells in tumor progression and metastasis, it is not surprising that targeting lymphocytes or hematopoietic stem cells (HSCs) with NPs has proven to be an exciting therapeutic option.^[^ 63 ^]^ Using this concept, NP‐based gene delivery to tumor‐associated cells can induce the transformation of the immunosuppressive TME into a toxic environment for cancer cells,^[^ 106 ^]^ which may potentially offer a universal approach to immunotherapy.^[^ 107 ^]^ Furthermore, NPs appear to be a promising tool to reprogram tumor‐associated cells such as fibroblasts, immune cells (macrophages), dendritic cells (DCs), blood endothelial cells (ECs), and lymphatic vasculators.^[^ 36 ^]^

It was indeed demonstrated that cancer‐associated fibroblasts (CAFs) can serve as cellular generators to produce immunosuppressive TME‐inhibitory proteins.^[^ 108, 109 ^]^ For example, traps, namely fusion proteins designed to bind tumor‐soluble factors such as cytokines, can be successfully delivered for cancer therapy by that approach. In one study, CXCL12 and PD‐L1 traps, when combined, resulted in enhanced T cell trafficking and the inhibition of liver metastasis as compared with either therapy alone.^[^ 108 ^]^

**Figure 5:**
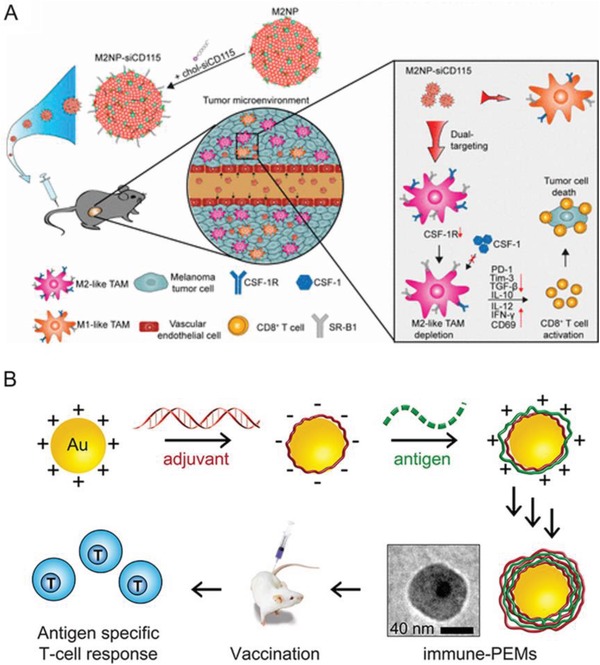
Nanoparticle‐mediated tumor‐associated cells reprogramming to enhance anti‐tumor functions of T cells. A) M2‐like TAM dual‐targeting nanoparticles (M2NP)‐based delivery of siRNA for CSF‐1R silencing and immune regulation via synergistic dual targeting of M2‐like TAMs in vivo. Reproduced with permission.^[^ 111 ^]^ Copyright 2017, American Chemical Society. B) Polyelectrolyte multilayers assembled entirely from immune signals on gold nanoparticle to activate APCs and to stimulate the formation of antigen‐specific CD8^+^ T cells both in vitro and in vivo. Reproduced with permission.^[^ 119 ^]^ Copyright 2015, American Chemical Society.

Myeloid‐suppressor cells (MDSCs) were suggested to be a major therapeutic barrier hindering the anti‐tumor function of T cells, but it was shown that polymeric micelles conjugated with 6‐thioguanine can inhibit the effect of MDSCs and stimulate the activity of T cells in a murine model.^[^ 110 ^]^ Similarly, the delivery of anti‐colony stimulating factor 1 receptor (CSF‐1R) siRNA to tumor‐associated macrophages (TAMs) in a melanoma mouse model increased CD8^+^ cytotoxic T cell function, by inhibiting immunosuppressive cytokines TGF‐β and IL‐10 and inducing immunostimulatory cytokines interferon‐γ (IFN‐γ) and IL‐12, proinflammatory mediators that links between innate and adaptive immunity. These therapeutic effects inhibited tumor growth by 87% and prolonged mouse survival^[^ 111 ^]^ (**Figure** **5**A). The combination of a polymeric‐liposomal gel system with TGF‐β inhibitor has been also found to stimulate the activity of natural killer (NK) cells against different cancer types and to increase immune response.^[^ 112 ^]^ Saeid et al. further demonstrated that the FDA‐approved ferumoxytol nanomicelle iron supplement can suppress tumor growth by stimulating pro‐inflammatory macrophage polarization at tumor sites.^[^ 113 ^]^

TAMs have been also targeted in vivo by the injection of 60 nm polysaccharide nanoparticles prepared from cholesteryl pullulan to facilitate T cell activation. The authors indeed demonstrated that their system stimulated anti‐tumoral CD8^+^ T cell response.^[^ 114 ^]^ In a recent study, a dual drug‐loaded NP formulation was shown to accumulate selectively within TAMs and to improve anti‐tumor efficacy in murine cancer models.^[^ 115 ^]^

The unique cell surface markers expressed by TME‐associated cells represent an enticing direction to pursue and can likewise serve to achieve targeted delivery.^[^ 105 ^]^ In one study, the expression of intracellular migration inhibitory factor from macrophages was effectively inhibited by the injection of siRNA‐loaded glucan NPs, resulting in CD4^+^ and CD8^+^ cell expansion at tumor sites and the promotion of anti‐tumor immunity, by inducing inflammatory cytokines such as tumor necrosis factor‐α (TNF‐α) and IL‐2.^[^ 116 ^]^

DCs are one of the major APCs, with a vital role in the communication between innate and adaptive immunity.^[^ 117 ^]^ Modulation of the DC antigens to cross‐presentation CD8^+^ T cells is critically needed for effective anti‐tumor response and considered to be an attractive TME target. Based on this fact, the similarity of different types of NPs of comparable size and shape to pathogens can be exploited in the development of novel cancer immunotherapy technologies, by stimulating the immune system to support T cells in tumor eradication.^[^ 118 ^]^ Zhang et al. thus developed polyelectrolyte multilayer (PEM) systems conjugated with surface immune signals (iPEMs) and self‐assembled on AuNPs for that purpose. These NPs were shown to activate APCs and to stimulate the formation of antigen‐specific CD8^+^ T cells both in vitro and in vivo. These findings clearly confirm that antigens can be presented in a way to enable the expansion of T cells with specificity for these antigens, resulting in the secretion of effector cytokines^[^ 119 ^]^ (Figure 5B). NP‐based systems can also be used in artificial antigen‐presenting cells (aAPC), to stimulate T cells and activate them against tumor cells. Microscale (5–10 µm) aAPCs were designed to present signals for three different purposes: to facilitate T cell recognition of the antigen, to stimulate T cell activation, and to modulate T cell response.^[^ 120 ^]^ These aAPCs were demonstrated to be highly potent in systemic injection, and would be expected to produce even superior results when used in combination with the next generation of cancer nanomedicines. For example, liposome NPs modified with dextran and encapsulated in pH‐sensitive ovalbumin were used to target the cytosol of DCs.^[^ 121 ^]^ This system successfully activated antigen‐specific cellular immunity through the MHC‐I pathway, and ultimately enhanced the activity of CD8^+^ T cytotoxic cells, serving to counter infection and to generate sustained memory T cells against recurrent infection.^[^ 122 ^]^

Furthermore, small virus‐sized particles (≤40 nm) can also act as tumor antigens and drain into tumor‐draining lymph nodes (TDLNs), where they are uptaken by DCs and then presented to T cells.^[^ 123 ^]^ However, the presence of immature/nonactivated DCs in TDLNs compromises the antitumor responses of T cell. Recently, nanocarriers of DC such as nanoparticle‐bound cytosine‐phosphateguanine (CpG) oligonucleotides promoted the maturation and activation of TDLN DCs in a melanoma model.^[^ 124 ^]^ These NP complexes were immediately uptaken by DCs and stimulated the release of the cytokines IL‐6 (exerts various pathological effects on autoimmunity and chronic inflammation) and IL‐12, resulting in potent antitumor immune responses.^[^ 125 ^]^ Similar results were achieved when different types of NPs (25–270 nm in diameter) were applied, including pyridyl disulfide‐, liposome‐, and gelatin‐based compounds.^[^ 124, 125 ^]^

In an epigenetics‐based study, the delivery of oncogenic miR‐155 mimics by biocompatible PEI NPs resulted in potent anti‐tumor effects in mice models, without disease progression 80 days after treatment. This therapy was associated with transcriptome‐wide changes in tolerogenic DCs, which were re‐programmed into immunostimulatory cells, resulting in the stimulation of robust T‐cell‐mediated anti‐tumor immunity.^[^ 67 ^]^ Furthermore, these results highlighted once more the usefulness of nanocomplexes (miRNAs with NPs) in reprograming the TME. Within this framework, the unique characteristics of NPs (size, shape, surface moieties) have been explored extensively to achieve superior conditions improving T cell programming, transfection, and therapeutic efficiency.^[^ 63, 73 ^]^ Representative examples of such NP systems are summarized in Table 3. It should be considered that nucleic acids comprise either ribose (RNA) or deoxyribose (DNA) and display additional properties such as hydrophilicity and negative charges allowing them to be efficiently encapsulated into NPs. Consequently, transient expression through nano‐systems may be among the best options currently available, and can be implemented in clinical trials as a novel form of effective immunotherapy.

Another effective approach is the normalization of the TME by targeting tumor‐associated vessels, which may decrease the efficacy of CAR‐T cell immunotherapy by lowering the antitumor immunity of T cells, or by reducing the immunosuppression level in the TME.^[^ 29 ^]^ A combination of antiangiogenic therapy and immunotherapy was recently recommended to enhance the effectiveness of adoptive immunotherapy,^[^ 126 ^]^ since abnormal vessels can directly kill T cells or thwart their immunosurveillance and trafficking.^[^ 127 ^]^ The “leaky” nature of tumor blood vessels is nevertheless also believed to support the delivery of nanotherapeutics to tumor sites through the enhanced permeability and retention (EPR) effect, which is influenced by the interplay between the NPs and the nature of the tumor.^[^ 128 ^]^ Consequently, targeting tumor‐associated vessels may lead to more potent therapeutic efficacy than targeting tumor cells directly. On this basis, new‐generation NPs with the addition of ligands to target specific antigens on vascular endothelial cells have been investigated. For instance, the peptide arginylglycylaspartic acid (RGD), an αVβ3 integrin ligand, was utilized to facilitate the uptake of doxorubicin NPs in the neo‐vasculature, so as to reduce tumor blood vessels and metastasis.^[^ 129 ^]^ Interestingly, chitosan NPs were found to effectively co‐localize and enhance siRNA delivery to both endothelial and tumor cells in vivo.^[^ 130 ^]^ Thus, NPs can increase therapeutic efficiency by stimulating replicative or anti‐replicative effects, stimulating antigen presentation, and can be exploited to optimize T cell genetic engineering.^[^ 33, 131 ^]^

**Figure 6:**
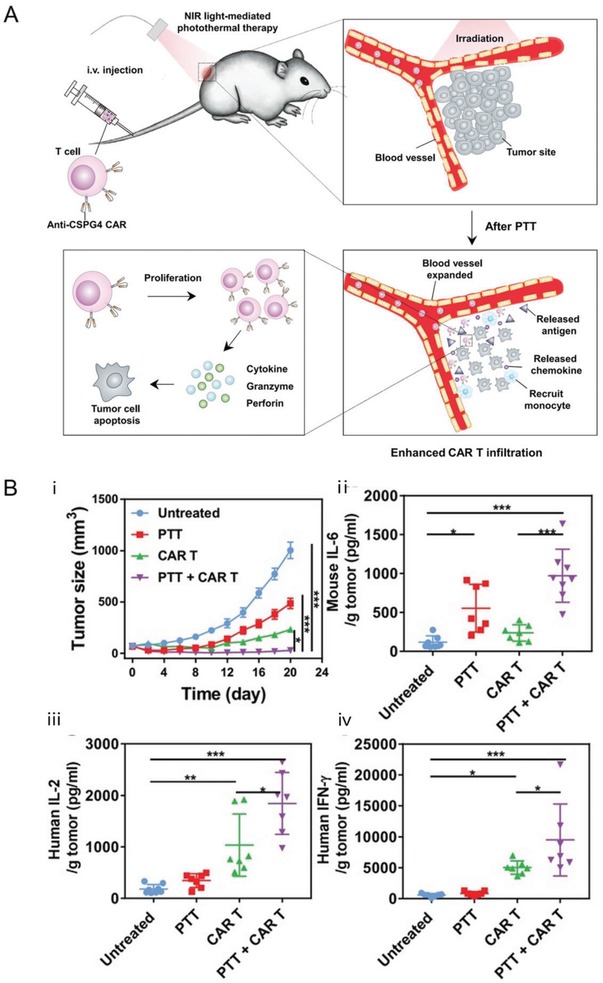
Nanoparticles can improve T cell therapy by remodeling the microenvironment created by solid tumors. A) Photothermal nanoparticles that generate heat under the near‐infrared (NIR) light irradiation can reduce the compact structure and interstitial fluid pressure, increase blood perfusion, release antigens, and promote the recruitment of endogenous immune cells in solid tumors. These effects can then cause enhanced infiltration and activation of tumor‐specific CAR‐T cells. B) Photothermal therapy combined with CAR.CSPG4^+^ T cells significantly suppressed the tumor growth up to 20 days as compared with control (i). Murine IL‐6 was increased after photothermal therapy (ii). Moreover, human IL‐2 (iii) and IFN‐ γ (iv) released by CAR T cells were also significantly increased, especially in the mice receiving the combined treatment groups. Reproduced with permission.^[^ 31 ^]^ Copyright 2019, John Wiley and Sons.

CAR‐T cell therapy has achieved remarkable progress in the treatment of blood cancers such as ALL. However, it faces challenges in treatment of solid tumors. A major obstacle to effective CAR‐T cell therapy against solid tumors is the microenvironment antagonistic to T cells. Nanotechnology approaches have been utilized to promote T cell therapy for solid tumors through preconditioning of solid tumors. Gu et al. proposed photothermal nanoparticles of poly(lactic‐*co*‐glycolic) acid (PLGA) loaded with indocyanine green (ICG), to precondition the solid tumor under the near‐infrared (NIR) light irradiation.^[^ 31 ^]^ The mild hyperthermia of the tumor reduced its compact structure and interstitial fluid pressure (IFP), increased blood perfusion, released antigens, and promoted the recruitment of endogenous immune cells (**Figure** **6**A). These effects promoted the penetration and therapeutic index of CAR‐T cells in solid tumors (Figure 6B). Stephan et al. demonstrated that targeted liposomes that deliver a combination of immune‐modulatory agents can remove protumor cell populations and simultaneously stimulate antitumor effector cells. This treatment created a therapeutic window of 2 weeks for tumor‐specific CAR‐T cell therapy against solid tumors.^[^ 132 ^]^

## 5 Nanoparticles Improve the Imaging Modalities for Tracking CAR and CAR‐T Cell Fates

While several new cell therapies involving CAR‐T cells have been approved or are currently in clinical trials for cancer immunotherapy, the strategies available to monitor CAR transgene cassettes and CAR‐T cells or their therapeutic effects are limited. Microtechologies such as chromium (Cr‐51) release assay, quantification of cytosolic enzymes (e.g., lactose dehydrogenase (LDH)), time‐lapse imaging, electrical impedance sensors, and micropatterning were used to measure and monitor CAR‐T cell cytotoxicity.^[^ 133 ^]^

Besides microtechnologies, noninvasive and reproducible diagnostic technologies such as single‐photon emission‐computed tomography (SPECT/CT), positron emission tomography (PET), and magnetic resonance imaging (MRI) can also serve to track the response to CAR‐T cell therapy. To this specific end, T cells were engineered to carry labeled antigens or receptors that could be monitored by SPECT/CT, for example, with indium‐111 (^111^In), to allow tracing of the transferred T cells in vivo.^[^ 134 ^]^ This approach was shown to provide good image resolution, although its applicability was limited by signal loss after 96 h (owing to isotope decay), such that its ability to track CAR expression in T cells was questioned. Furthermore, while labeling is not reported to affect cellular proliferation, it may nevertheless interfere with the activity of T cells.

In in vitro and in vivo prostate cancer models, SPECT/CT imaging with 99mTcO4 radiotracer also allowed tracking of CAR‐T cells.^[^ 135 ^]^ This was achieved using a retroviral vector to introduce norepinephrine receptors or human reporter gene sodium/iodide symporters (hNIS) into T cells. This methodology provided radiotracer kinetics in transduced T cells and confirmed the utility of this non‐invasive system for CAR‐T cell tracing in patients. Unfortunately, the use of viral vectors and the uptake of tissue‐specific tracers by hNIS may hinder the application of this system to trace T cells directed against thyroid and stomach tumors.

Based on the concept of co‐expression within the same type of cells, CAR and a luciferase reporter were co‐expressed with a herpes simplex virus thymidine kinase 1 (HSV1tk) reporter. This approach allowed the imaging of CAR‐T cells via both bioluminescence imaging and non‐invasive PET.^[^ 136 ^]^ In the same spirit, the co‐expression of somatostatin receptor type 2 (SSTR2) and intracellular adhesion molecule‐1 allowed CAR imaging via PET/CT using a gallium‐68‐labeled octreotide analogue.^[^ 137 ^]^ Unfortunately, co‐expression strategies are limited by different entails. For instance, the first approach mentioned turned out to enhance immune‐mediated recognition due to the use of the immunogenic HSV1tk viral protein.^[^ 138 ^]^ The second approach was limited by poor sensitivity^[^ 139 ^]^ to octreotide analogs, since SSTR2 is not only present in T cells but also in other immune cells.^[^ 140 ^]^

PET‐based T cell imaging can likewise potentially serve to monitor tumor‐infiltrating T cells, and to visualize the activity of T‐lymphocytes in response to immunotherapy. ImmunoPET imaging can serve to monitor the fate of adoptive T cells and to provide information on events involving monoclonal antibodies in vivo (e.g., their accumulation in normal tissues, tumor targeting, and quantitative variations) and, as a result, may allow the optimization of their immunotherapeutic efficacy.^[^ 141 ^]^ MRI may also potentially serve for T cell tracking in vivo, albeit a consistent technique to track non‐phagocytic T cells and the development of an MRI contrast agent suitable for that purpose are required.

Interestingly, the unique features of NPs may allow them to address the aforementioned limitations in several ways. For instance, adoptively transferred T cells were successfully tracked using radiolabeled PEGylated AuNP‐^64^Cu, loaded in the CD19^+^ CAR‐T cells by electroporation, and then imaged by the µPET/CT technique.^[^ 142 ^]^ These results indicated that PEGylated AuNP‐^64^Cu is useful as a radiotracer for the in vivo monitoring of T cell immunotherapy. These innovative probes have allowed the investigation of changes in T cell functions, location, and numbers,^[^ 143 ^]^ enabling T cell tracking in different hematopoietic organs or within tumors. Furthermore, immuno PET imaging can be improved with tracers such as radiolabeled NPs or nanobodies, by reducing the radiation dose and increasing the target‐to‐background ratio.^[^ 144 ^]^ Radiolabeled nanoparticles such as iron oxide nanoparticles (IONPs) have shown promise to overcome the limitations of the individual PET or MRI techniques, by offering more specific radiolabeling for the next generation of imaging agents, and could lead to significant improvements in tracking adoptively transferred T cells in vivo.^[^ 145, 146 ^]^ For instance, Liu et al. developed PEG‐coated superparamagnetic IOPC‐NH_2_. These NPs were shown to label both animal and human T cells efficiently (over 90%), without affecting their behavior, and could be detected by MRI in vivo. Most importantly, this system did not require the viral transfection vector HIV‐1 transactivator peptide, or non‐viral based vectors such as electroporation. Furthermore, IOPC‐NH_2_ can serve as a potent contrast agent to track a wide range of cells, including CAR‐T cells.^[^ 147 ^]^

Besides monitoring CAR‐T cell therapy, the group of Sengupta was able to monitor the efficacy of immunotherapy in vivo by designing 2‐in‐1 reporter NPs.^[^ 38 ^]^ The PD‐L1 reporter NPs produced were found to stimulate the level of activated‐caspase‐3 in the B16‐F10 melanoma tumor model, leading to promoted T cell trafficking activity at tumor sites, in addition to the production of a strong fluorescence signal.^[^ 38 ^]^ Accordingly, these types of reporter NPs have the capacity to both deliver and report on therapeutic efficiency in real time.

## 6 Conclusions

The limitations of the genetic tools available to engineer T cells have motivated researchers to develop novel methods. Furthermore, CAR‐T cell manufacturing issues such as harvesting a sufficient number of T cells, the selection of T cell starting phenotypes, toxicity, disappointing standard T cell checkpoint inhibitory markers, patient heterogeneity, as well as the presence of immuno‐suppressor cells and abnormal vessels also limit the use of CAR‐T cell immunotherapy. While certain aspects of CAR construct and vector designs affect the characteristics of CAR‐T cell products, their expansion kinetics, and activity, various intuitive approaches are being explored to improve the therapeutic efficacy of CAR‐T cells.

**Figure 7:**
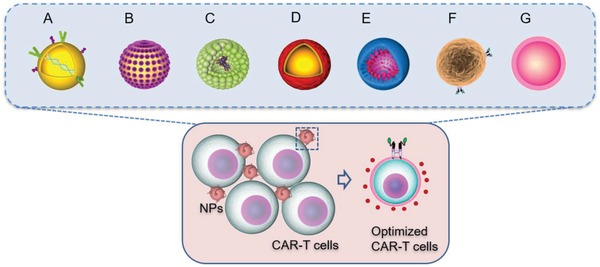
Depiction of representative nanotechnology‐based strategies to optimize CAR‐T cell therapy: A) polymeric NPs to optimize gene delivery and the in situ generation of CARs; B) surface‐conjugated nanogels to improve T cell expansion; C) liposome‐encapsulated proteins leading to T cell activation; D) NPs preconditioning tumor to promote T cell penetration and antitumor activity; E) NPs coupled with checkpoint inhibitors to induce CAR‐T cell trafficking; F) synthetic antigen receptor to overcome immunosuppressive tumor‐associated cells and vessels by different mechanisms; G) radio‐labeled NPs for CAR‐T cell tracking.

Among these, NPs can address the engineering requirements of adoptive T cells and seem particularly attractive as optimization methods (**Figure** **7**). While NP‐based cancer immunotherapy is at an early stage of development, it holds remarkable potential. It was indeed confirmed that the use of engineered, versatile, NP‐based platforms can lead to significant improvements in the design and therapeutic efficacy of engineered adoptive T cells through i) the generation of therapeutically effective CAR transgene cassettes, ii) the delivery of antigens selectively to target sites, thus decreasing their toxicity, iii) the modulation of the activity of CAR‐T cells by adapting immune checkpoints, iv) overcoming immunosuppressive tumor‐associated cells and vessels, and vi) tracking the fate of therapeutic CAR‐T cells and relevant molecular markers.

While various NP systems have been successfully used to address the issues identified above, unintentional gene transfer into off‐target cells, and the immunogenic (e.g., inflammatory and antigenicity) characteristics of these NPs still represent major challenges in their design. In addition to design principles, pharmacokinetics, and systemic toxicity, it is critical to tune the nanoparticle size, shape, and surface charge, in order to minimize unwanted nanoparticle‐based immunization responses. Although small nanoparticles (10–40 nm in diameter) can migrate successfully within and between immune cells, the potential toxicity remains to be critically addressed in the majority of these studies. Short‐term and long‐term studies with specific NPs are also needed with both human cells and living animal models, to determine the behavior of these particles in immune and cancer cells. Consequently, the judicious selection of novel NPs with superior characteristics (fully biocompatible, highly stable after preparation, and useful for frequent administration) and the design of their functionality (stimuli‐responsive to pH, temperature, specific enzymes, etc.) should be most helpful to minimize their toxicity and to optimize the therapeutic efficacy of CAR‐T cell therapy.

## Conflict of Interest

The authors declare no conflict of interest.
